# A Federal Report on Veneral Diseases

**Published:** 1916-09-16

**Authors:** 


### A FEDERAL REPORT ON VENEREAL DISEASES.

### Drastic Proposals

On July 5 the Committee appointed to investigate the causes of death and invalidity throughout the Commonwealth of Australia presented their report on venereal diseases, with recommendations for combating them, to the Minister for Customs. The Melbourne City Council immediately moved in the matter by approaching the Government of Victoria. The matter is a national one; but each State will manage it in its own way. The Federal Government has decided that it will grant £15,000 annually towards the fight for the eradication of these diseases, to be divided among the six States, according to population. The gist of the report is appended:—

The Committee states that syphilis is responsible for more diseases, more incapacity, and more misery than any other single agent or cause. The number of deaths among children in the Commonwealth as a result of the presence of disease in either of their parents was 3,326 during 1914. The Committee urges the introduction of special legislation with the object of ensuring that every person suffering from venereal disease shall undergo efficient treatment and continue under such treatment until they are no longer infected. The legislation recommended would provide :—

(1) That it be unlawful for any person other than a legally qualified medical practitioner to treat any case of venereal disease. (2) That any person suffering from venereal disease be required to consult a medical practitioner, and to place himself or herself under treatment by that practitioner. (3) That every medical practitioner shall notify such cases as come under his professional notice, and shall report to the health authorities if the patient does not consult him again within an approved period. (4) That every practitioner attending a case of venereal disease shall warn his patient of the legal penalties for communicating his disease to others, and against marrying before he has received a certificate of cure. (5) That due responsibility be placed on parents or guardians of infected persons under sixteen to see that they are placed under proper treatment. In the case of a patient failing to obey an order to place himself under the care of a medical practitioner, the Minister may cause that patient to be interned in a hospital until cured or declared to be no longer infected; provided that any person so detained shall have the right of appeal to a judge of the Supreme Court, or to a police magistrate, but not so as to interfere meanwhile with the order of the Minister. These provisions should also apply to prisoners under sentence. (6) That it be an offence knowingly to infect any person with venereal disease, or to do any act likely to cause such infection. (7) That all advertisements concerning remedies for venereal diseases be prohibited. (8) That if an infectious person persists in the intention to marry, despite the warning of his or her medical attendant, a communication made *bona fide* by the medical practitioner to the other party to the marriage on the matter shall be privileged, and that, if a person marries in the infective stage, without giving information to the other party, such act shall be a ground for divorce.

Western Australia is the only State that has any real or vigorous legislation on this matter. The Chief Secretary of South Australia has stated that the Government have resolved to establish night clinics at Adelaide Hospital. The Victorian Minister for Health has not yet decided in what form the necessary provisions will be introduced, but they will be on the lines of the West Australia Act and the recommendations given above.

Other recommendations by the Committee relate to treating infected seamen at every chief port, the desirability of an educational movement whereby every boy on attaining the age of fifteen years should be taught the lesson of clean living and continence by his schoolmaster. This latter proposal has evoked much opposition, especially from the Churches.

